# An Automated Framework for Pose-Sequence Analysis of Spontaneous Movements in Preterm Infants

**DOI:** 10.1109/OJEMB.2026.3735159

**Published:** 2026-09-17

**Authors:** Sofia Sigismondi, Sara Uccella, Chiara Tacchino, Luca Ramenghi, Lino Nobili, Francesca Odone, Maura Casadio, Matteo Moro

**Affiliations:** Department of Informatics, Bioengineering, Robotics and Systems Engineering (DIBRIS)University of Genova 16126 Genova Italy; IRCCS Istituto Giannina Gaslini Hospital 16147 Genova Italy; ^3^ Department of Neurosciences, Rehabilitation, Ophthalmology, Genetics, Maternal and Child Health (DINOGMI)University of Genova 16126 Genova Italy; IRCCS Istituto Giannina Gaslini Hospital 16147 Genova Italy; Department of Informatics, Bioengineering, Robotics and Systems Engineering (DIBRIS)University of Genova 16126 Genova Italy; ^6^ Machine Learning Genoa (MaLGa) Center 16146 Genova Italy; Department of Informatics, Bioengineering, Robotics and Systems Engineering (DIBRIS)University of Genova 16126 Genova Italy; ^8^ IRCCS Istituto Giannina Gaslini Hospital 16147 Genova Italy; Department of Informatics, Bioengineering, Robotics and Systems Engineering (DIBRIS)University of Genova 16126 Genova Italy; ^10^ IRCCS Istituto Giannina Gaslini Hospital 16147 Genova Italy; ^11^ Machine Learning Genoa (MaLGa) Center 16146 Genova Italy

**Keywords:** Cerebral palsy, developmental delay, human pose estimation, preterm infants, spontaneous movement analysis

## Abstract

*Goal:* Spontaneous movement analysis is crucial for early neuromotor assessment, yet objective, unobtrusive, and scalable tools remain limited. This study investigates an automated video-based framework for characterizing spontaneous movements in preterm infants. *Methods:* Videos of 142 infants at term-equivalent age were analyzed using markerless two-dimensional pose estimation. Pose configurations were clustered into a pose dictionary, and recordings were represented as temporal sequences of key poses from which movement parameters were derived. Infants with typical development (TD) were compared with those with cerebral palsy (CP) and developmental delay (DD) stratified into low and high motor severity. *Results:* Compared with TD, CP and DD–high showed significantly lower pose transition (median: 0.38 and 0.42 vs 0.50 transitions/s) and accumulated fewer unique poses over time, while CP also showed lower pose variability (median: 0.79 vs 0.87). *Conclusions:* The framework characterizes pronounced neuromotor impairments and may provide an objective approach for early motor characterization.

## I. Introduction

Spontaneous motor activity is one of the earliest indicators of neuromotor organization in newborns. Quantitative analysis provides insight into early neurodevelopment and deviations from typical trajectories [1], [2]. Preterm birth, accounting for approximately 10% of births worldwide, is associated with increased risk of adverse neurodevelopmental outcomes, including motor impairments [3]. Given the high brain plasticity of early infancy, identifying atypical motor patterns may enable timely intervention and improve long-term outcomes [4], [5].

The General Movements Assessment is the clinical standard for evaluating spontaneous movements in early infancy [4], [6]. Through direct or video-based qualitative observation of infants’ movements, trained clinicians can identify infants at risk of neuromotor impairment. However, its subjective nature and reliance on specialized training limit scalability [7]. Marker-based systems provide detailed pediatric kinematic measurements [8], [9], whereas markerless video-based approaches offer a non-invasive and scalable alternative [10], [11], [12]. Automated infant movement analysis has evolved from frame-differencing and optical flow [13], [14], [15], [16], [17] to pose-estimation approaches that track anatomical keypoints and analyze pose-derived features using machine- and deep-learning models [18], [19], [20], [21], [22]. Pose-derived entropy has quantified movement complexity and its association with motor outcomes [23], [24], while RGB-D sensors and benchmark 3D datasets enable more detailed representations [25], [26]. Nevertheless, many models provide predictions or latent features that are not easily translated into clinically meaningful movement descriptors, potentially limiting clinical adoption [27]. The present framework addresses this limitation by deriving pose-based descriptors related to observable aspects of movement organization.

We propose an automated pipeline for characterizing spontaneous movements in preterm infants from 2D videos. The framework represents movement as temporal sequences of key poses derived from a data-driven dictionary of body configurations [28]. Quantitative metrics characterize motor activity and differences across neuromotor outcomes. Preliminary work identified abnormal patterns using five keypoints (the nose, hands, and feet) [29]. Here, 15 keypoints provide full-body configurations with greater anatomical detail. The framework is evaluated in infants with cerebral palsy (CP) and developmental delay (DD) stratified by motor severity.

## II. Materials and Methods

### A. Dataset

The dataset consists of video recordings of spontaneous movements in preterm infants collected at the Istituto Giannina Gaslini in Genoa (Italy). Recordings were acquired at Term Equivalent Age (TEA), corresponding to 40 weeks of gestational age. Videos were recorded using a Canon Legria HF R37 RGB camera at 25 frames per second (fps) with a spatial resolution of 1920 × 1080 pixels. The camera was mounted above a cradle to capture the infant's full body from a fixed overhead perspective (Fig. 1), at a height of approximately 1-1.5 meters from the infant. During acquisition, infants were in a supine position, awake, and spontaneously moving. Video segments were excluded if movements were potentially influenced by external factors (e.g., crying, pacifier use, or clinical maneuvers) or if the body was partially occluded by clinical equipment. The average recording duration was 5.6 ± 3.4 minutes.

**Fig. 1.**
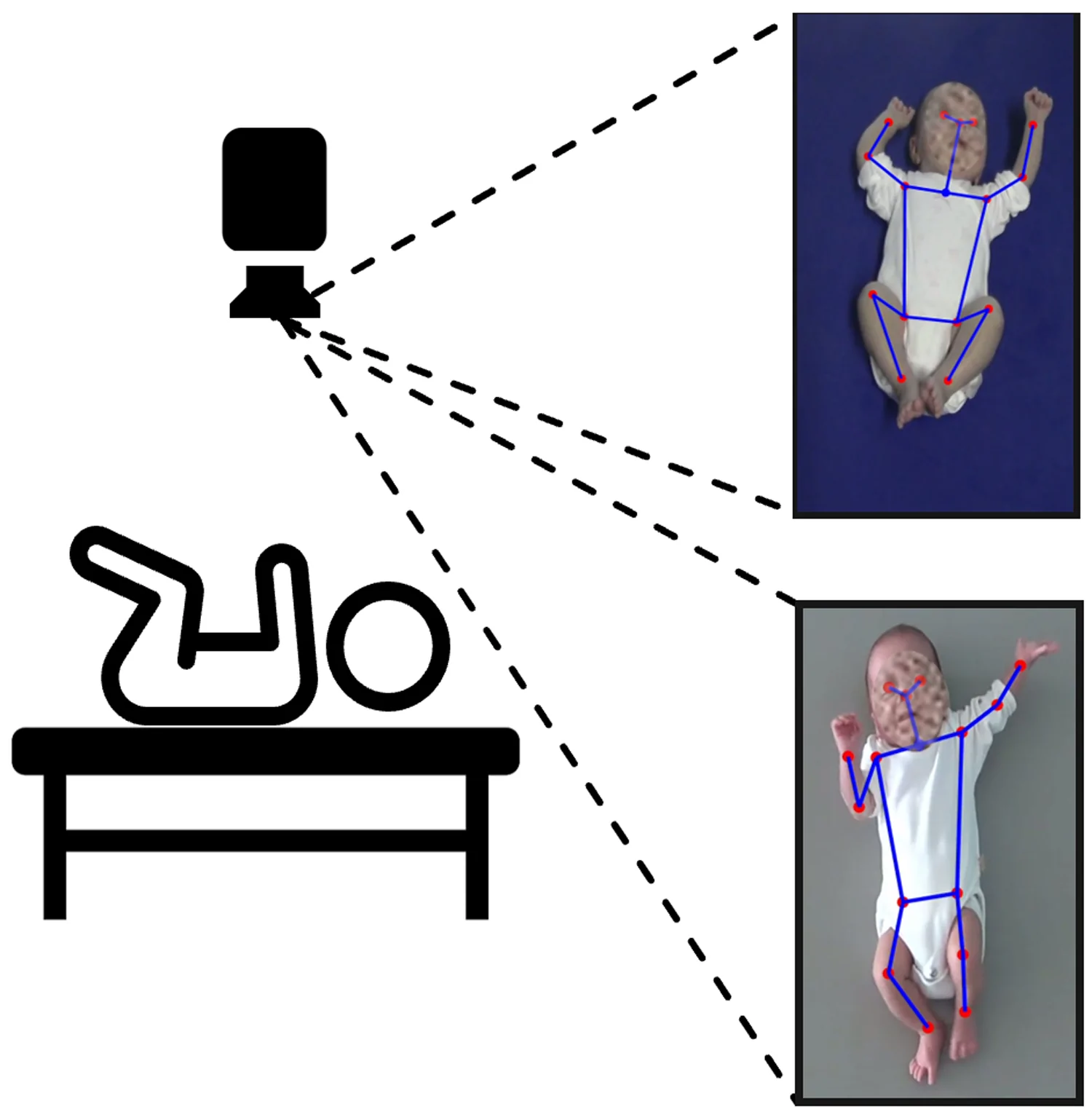
Overview of the acquisition setup and camera positioning, together with representative frames illustrating the detection of the 15 extracted 2D keypoints.

Infants were eligible if they were born before 32 weeks of gestation, had birth weight below 1500 g, were clinically stable at the time of recording, and had no recent sedation or ongoing respiratory support. The study timeline is summarized in Fig. 2. Among the enrolled infants, 88 showed typical neurodevelopment, 40 were diagnosed with developmental delay (DD), and 14 with cerebral palsy (CP). Neuromotor outcome was defined using clinical data collected during routine care [30]. All infants underwent brain MRI at TEA (T1 and T2 sequences) to identify structural abnormalities associated with neuromotor risk [31], [32] and were enrolled in a longitudinal follow-up program with clinical evaluations up to 30 months of corrected age, including standardized assessments such as the Bayley Scales of Infant and Toddler Development and the Griffiths Developmental Scales [33], [34], [35].

**Fig. 2.**
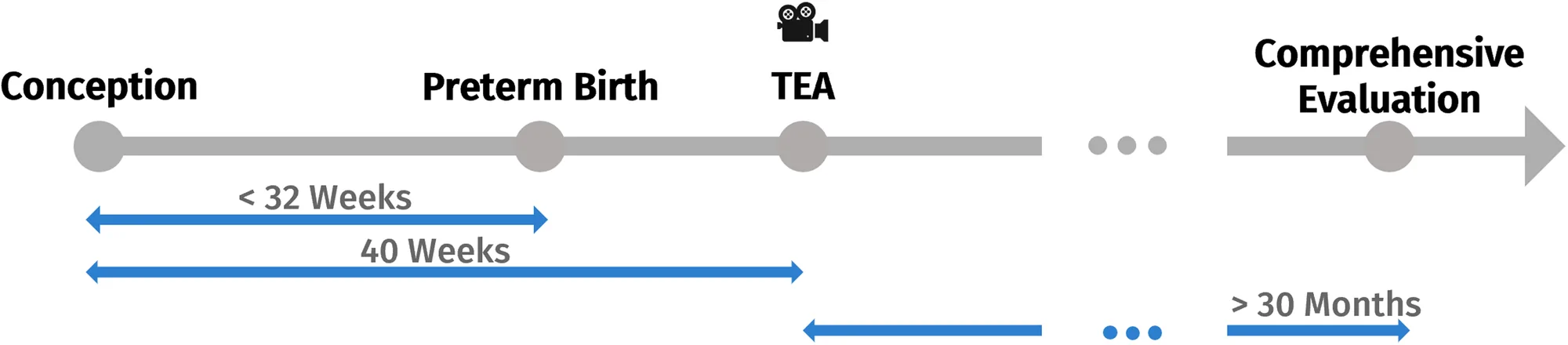
Overview of the experimental timeline. The study populations include preterm infants born before 32 weeks gestational age. Video recordings were acquired at Term-Equivalent Age (TEA). The comprehensive clinical ground truth evaluation was performed after 30 months of age.

Given the heterogeneity of DD, infants were stratified into two subgroups using the gross-motor subscale of the Griffiths Developmental Scales, where higher scores indicate better motor performance. A cut-off score of 80, selected based on the published interpretation of Griffiths III scores, was used to define two subgroups: DD–low impairment (scores ≥80) and DD–high impairment (scores <80). This value corresponds to the boundary between the “below average” range (80–89) and the lower interpretative categories [36], [37]. Because dichotomization may reduce the information provided by a continuous measure, complementary regression analyses used the gross-motor score continuously. DD–low showed higher gross-motor scores (mean ± SD: 86.3 ± 3.13; range: 80–92) than DD–high group (71.6 ± 7.65; range: 54–79). Demographic characteristics are reported in Table I.

**TABLE I:** Demographic Characteristics

	TD	DD-low	DD-high	CP	p-value
*Sex* *(F/M)*	55/33	14/6	7/13	8/6	0.175
*GA*	29.6 ± 1.9	28.4 ± 2.19	28.8 ± 1.8	28.5 ± 2.8	0.116
*BW*	1289 ± 307	1091 ± 241	1141 ± 265	1072 ± 272	0.004

Demographic characteristics including Gestational Age (GA), Birth Weight (BW) and number of Female (F) and Male (M) at Term-Equivalent Age (TEA)

Group differences were assessed using the following statistical tests: gestational age was compared using the Kruskal–Wallis test due to non-normal distribution, birth weight using one-way analysis of variance (ANOVA) with Tukey post-hoc tests, and sex distribution using the chi-square test. Sex distribution and gestational age did not differ significantly across groups. Birth weight differed significantly across groups (p = 0.004) with TD showing higher birth weight than infants with CP (p = 0.050), and DD-high impairment (p = 0.032).

Although birth weight is a well-established predictor of neuromotor risk and the observed group differences were therefore expected, it does not provide sufficient discriminatory power to define or stratify clinical outcomes on its own. For this reason, demographic variables were included for descriptive purposes, while the focus of the study was on movement patterns. The study protocol and the informed consent signed by the parents were approved by the Giannina Gaslini Hospital Institutional Review Board (protocol number: IGGPM01 20/06/2013).

### B. Pose Estimation

The video frames of each infant were processed using RTMPose for 2D pose estimation [38], extracting the spatial coordinates of 15 anatomical keypoints (Fig. 1), including bilateral ankles, knees, hips, shoulders, elbows, and wrists, together with the nose and eyes. RTMPose was selected based on preliminary testing on neonatal recordings and recent evidence supporting its use in neonatal pose estimation [39] and was employed using a model pretrained on the COCO dataset. Post-processing steps were applied to improve the robustness and temporal consistency of the extracted trajectories following the procedure described in [19]. Keypoints with confidence scores below 0.55 were treated as missing data; gaps shorter than 50 frames were interpolated using PCHIP, while longer gaps were retained as missing. Trajectories were then filtered using a zero-phase low-pass Butterworth filter with a cut-off frequency of 10 Hz to reduce noise [19], [38], [39].

### C. Spatial Normalization of 2D Keypoint Coordinates

To enable comparison across infants with different body size, camera distances and perspectives, the extracted 2D coordinates, originally expressed in pixels, were spatially normalized. A scale factor was computed as the mean trunk length over the entire video, defined as the Euclidean distance (in pixels) between the shoulder and hip midpoints. This value was used to rescale all the keypoint coordinates to a common reference length. Subsequently, for each frame, the trunk axis, defined by the line connecting the shoulder and hip midpoints, was rotated to align with the vertical axis. Finally, all coordinates were translated so that the midpoint of the shoulders was centered at the origin.

### D. Dictionary of Key Poses

To capture overall postural patterns while reducing temporal redundancy and computational load, video sequences were temporally downsampled to one frame per second before clustering. Inspired by bag-of-words models used in text analysis [40], k-means clustering was applied to the normalized 2D pose coordinates of all infants, grouping similar pose configurations into clusters. K = 20 was selected using the elbow method [41], and cluster inspection as a compromise between pose granularity, compactness, and interpretability, as lower K values suggested by other validity criteria merged anatomically distinct postural configurations. For each cluster, the centroid was mapped to the most similar real pose configuration in the dataset, identified using the Euclidean distance to obtain a representative and physically plausible key pose. The resulting set of K poses defined the key-pose dictionary. Each normalized pose was then assigned to the nearest dictionary element based on Euclidean distance, transforming each infant's video into a discrete temporal sequence of poses ${{\mathbf{P}}_s} = [ {{{p}_{s1}},{{p}_{s2}},\ldots,{{p}_{s{{T}_s}}}} ]$ where ${{\mathbf{P}}_s}$ denotes the pose sequence associated with infant *s*, ${{p}_{st}} $ the key pose assigned at time *t*, and Ts the length of the video for infants *s*.

This discrete representation was subsequently used to compute quantitative motion-related parameters (Fig. 3).

**Fig. 3.**
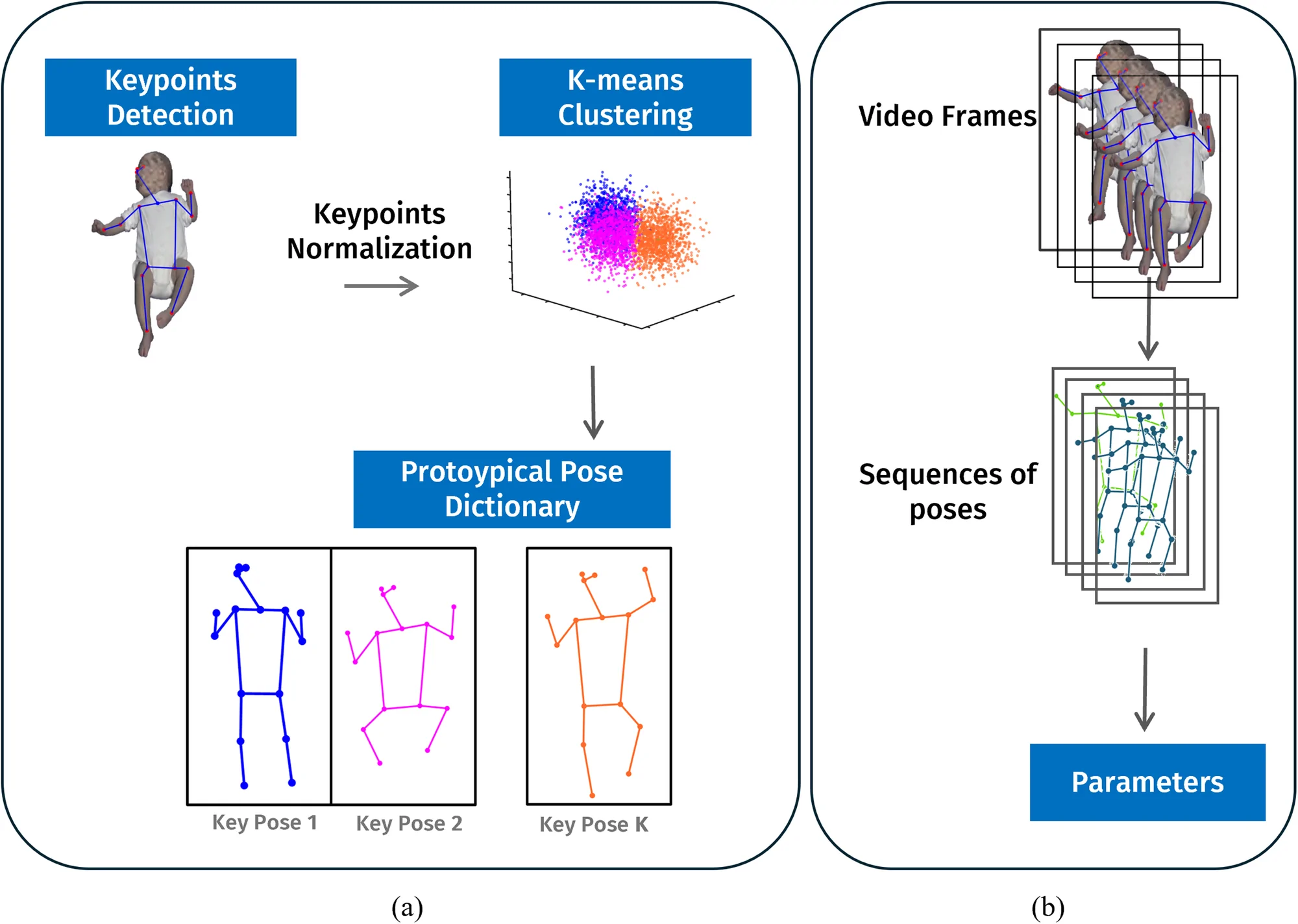
Overview of the proposed processing pipeline. (a) Extraction of normalized 2D pose configurations from infant videos. The normalized pose coordinates are subsequently used for unsupervised clustering in a low-dimensional embedding space to identify key poses. The resulting clusters define a pose dictionary, whose elements correspond to representative key poses. (b) Each video is encoded as a discrete temporal sequence of key poses, which is used for motion parameter computation.

### E. Parameters

Normal motor patterns are evaluated in terms of complexity and variability, reflecting the spatial diversity and temporal evolution of spontaneous movements [42]. Accordingly, three framework-specific metrics were extracted from the pose sequences to describe complementary aspects of the computed pose sequence: transition dynamics, qualitative variability, and repertoire expansion over time.

#### 1) Pose Transition Rate

The number of transitions between distinct key poses divided by video duration, quantifying how frequently an infant changes pose per unit time independently of the poses adopted.

#### 2) Index of Qualitative Variations (IQV)

Following the formulation described in [43] the IQV measures the diversity of pose usage over time and is defined as (1).

\begin{equation*}
IQV = \frac{{K(1 - \mathop \sum \nolimits_{j = 1}^K {{f}_j}^2)}}{{K - 1}} \tag{1}
\end{equation*}

Where ${{f}_j}$ denotes the relative frequency of the j-th key pose in the sequence and K the total number of poses in the dictionary. The IQV approaches zero when the pose sequence is dominated by a single pose, indicating low qualitative variability. Conversely, the IQV reaches its maximum value of 1 when the pose frequencies are uniformly distributed across all the K poses in the dictionary, reflecting high movement variability.

#### 3) Cumulative Unique Pose Count

To assess the expansion of the motor repertoire, we introduced the number of unique poses observed within a progressively increasing temporal window starting from the beginning of the recording. The window length was increased in 20-s steps starting from 20 s. For each recording, the parameter was computed up to the available recording duration; however, group-level analyses were limited to windows up to 180 s to ensure a sufficient number of recordings at each window length. This choice balances temporal resolution and clinical interpretability: shorter windows may not capture sufficient movement diversity, whereas longer windows may obscure relevant changes in the movement repertoire. Formally, the number of unique poses is defined as (2).

\begin{equation*}
{{N}_{{{p}_{s\ }}}}\left( {{{t}_{{{w}_j}}}} \right) = unique\left( {\left\{ {{{p}_{{{s}_1}}},\ {{p}_{{{s}_2}}},\ \ldots,\ {{p}_{{{s}_{twj}}}}} \right\}} \right) \tag{2}
\end{equation*}

where ${{t}_{{{w}_j}}}$ denotes the *j*-th time window defined as

\begin{equation*}
{{t}_{{{w}_{j\ }}}} = {{t}_0} + j*\ {{t}_{{{w}_{\min }}}}\quad j = 0,1, \ldots,\left\lfloor {\frac{{{{T}_s} - {{t}_0}}}{{\ {{t}_{{{w}_{\min }}}}}}} \right\rfloor . \tag{3}
\end{equation*}

with ${{t}_{{{w}_{\min}}}} = 20\;\mathrm{s}$ representing the temporal sampling interval, and $ {{T}_s}$ denoting the total duration of the video for infant *s*.

### F. Statistical Analysis

Normality was assessed using the Shapiro–Wilk test. As distributions were non-normal, non-parametric tests were used. The primary hypothesis was reduced movement activity and variability in infants with motor impairment. Three planned comparisons contrasted TD with DD–low, DD–high, CP; impairment groups were not compared with each other. Bonferroni correction was applied to the three planned comparisons, and adjusted p-values are reported.

Pose transition rate and IQV were compared using Wilcoxon rank-sum tests. Cumulative number of unique poses was analyzed using a Poisson generalized linear mixed-effects model with group, time window, and their interaction as fixed effects, and a subject-specific random intercept. The model included all available observations without imputation and accommodated unequal recording durations.

To further examine the relationship between motor severity and movement dynamics within the developmental delay cohort, exploratory linear regressions were performed using the gross-motor Griffiths score as a predictor, with pose transition rate and IQV as dependent variables in separate models. Statistical significance was set at p < 0.05.

## III. Results

Compared with typically developing peers, infants with CP (p = 0.003) and DD-high (p = 0.041) exhibited significantly reduced pose transition rates. In contrast, DD-low infants did not differ from typically developing infants (Fig. 4(a)).

**Fig. 4.**
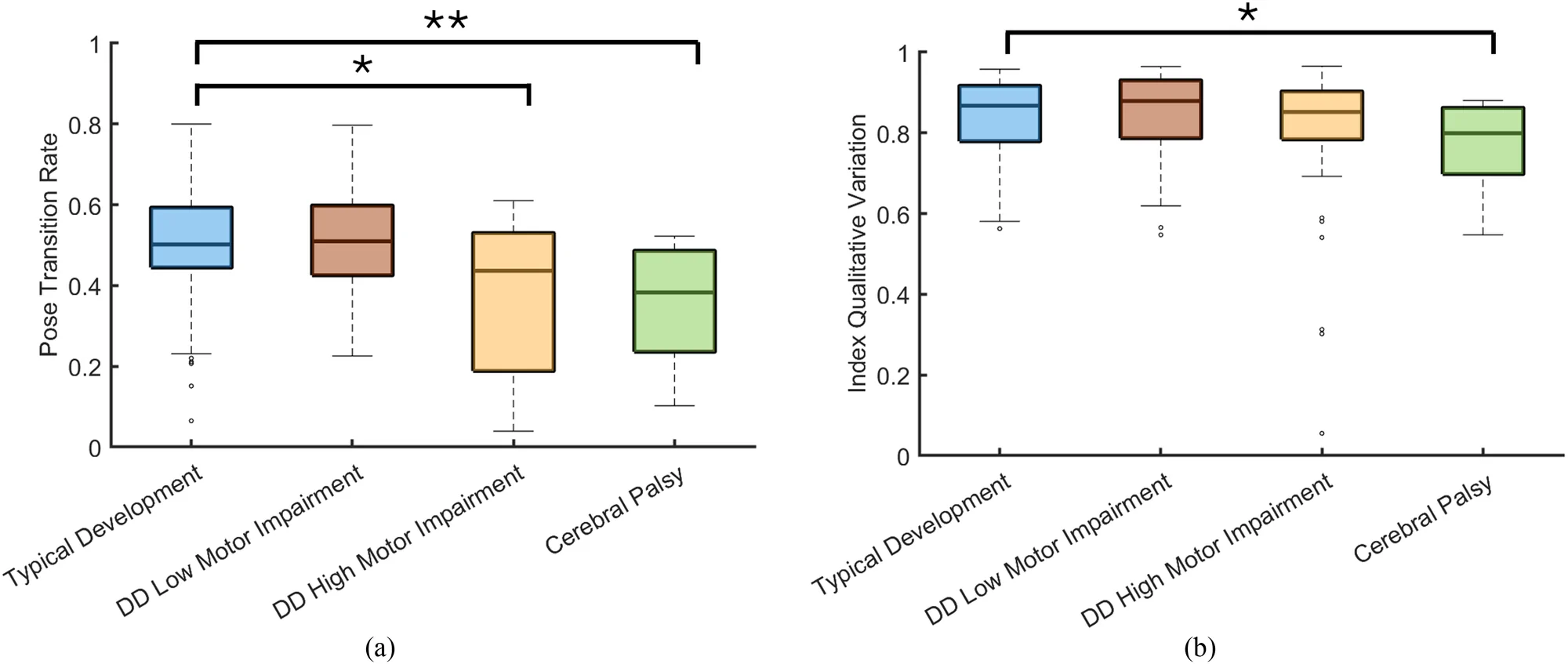
The boxplots from left to right show the distribution of (a) pose transition rate and (b) Index of Qualitative Variation across infants with typical development, DD with low motor impairment, DD with high motor impairment and CP. The central line indicates the median value; box limits correspond to the first and third quartiles; whiskers extend to the most extreme data points within 1.5 times the interquartile range. * denotes statistically significant differences between groups (*p < 0.05, ** p < 0.005).

Infants with CP exhibited lower IQV values compared to infants with typical development (p = 0.035, Fig. 4(b)), indicating that their movement patterns are dominated by a limited subset of poses rather than being evenly distributed across the broader repertoire available in the pose dictionary. No significant differences were observed between the DD subgroups and typically developing infants.

As an exploratory analysis, we further investigated the developmental delay (DD) cohort. Linear regression analysis revealed a positive association between the gross-motor Griffiths score and pose transition rate (β = 0.007, p = 0.028). The model explained 12% of the variance in pose transition rate (R^2^ = 0.12) (Fig. 5(a)). Also, an exploratory linear regression analysis was also performed to assess the relationship between motor severity and IQV. The gross-motor Griffiths score was positively associated with IQV (β = 0.01, p = 0.002), with the model explaining 22% of the variance (R^2^ = 0.22) (Fig. 5(b)).

**Fig. 5.**
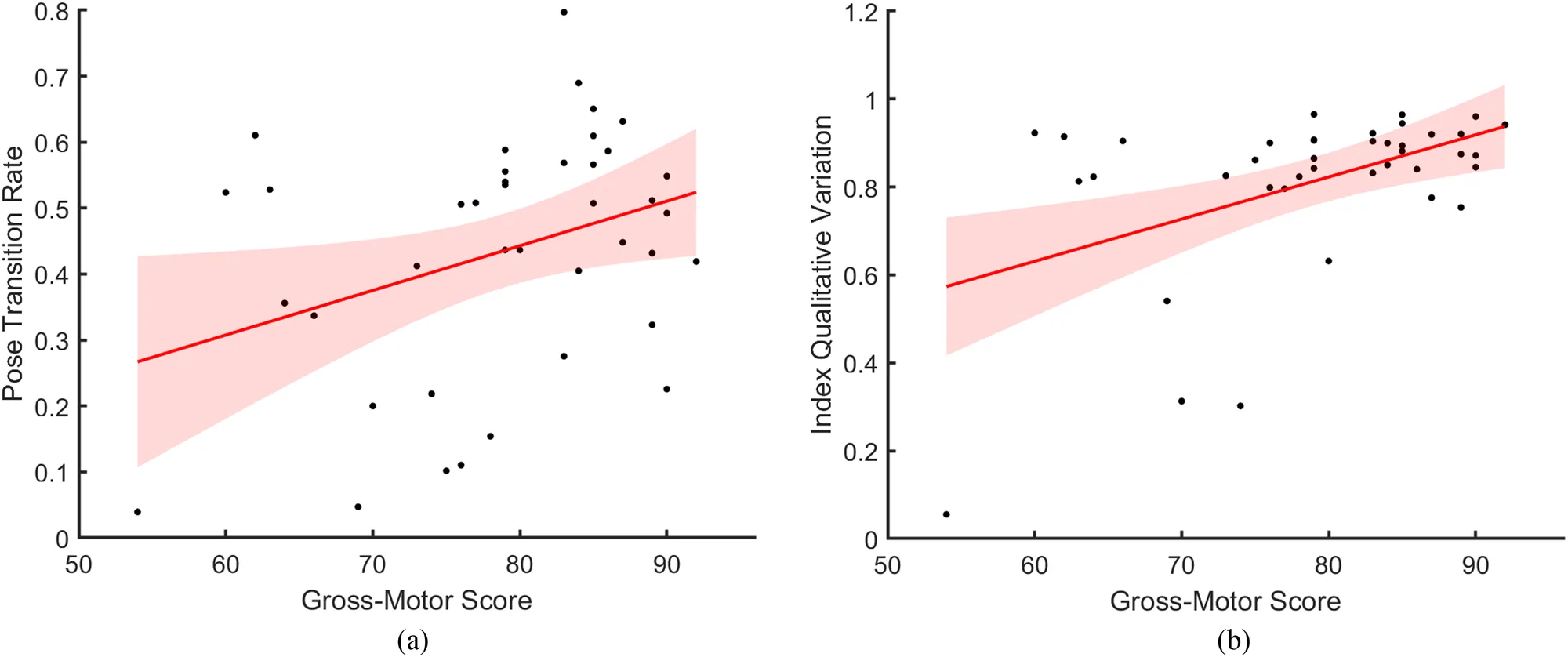
Linear regression analysis between gross-motor score and (a) pose transition rate and (b) Index Qualitative Variation within the developmental delay (DD) group. Each point represents an individual infant. The solid line denotes the fitted linear regression model (β = 0.007, p = 0.028; β = 0.01, p = 0.002 for (a) and (b) respectively).

The temporal evolution of the cumulative unique pose count over the first 180 seconds was analysed using a Poisson generalized linear mixed model (Fig. 6). A significant main effect of group was observed (χ^2^(3) = 14.4, p = 0.001), indicating differences in repertoire size across motor severity levels. A significant effect of time was also found (χ^2^(8) = 358.8, p < 0.001), reflecting the progressive increase in unique poses over time. No significant group-by-time interaction was detected (χ^2^(24) = 12.2, p = 0.978), suggesting similar temporal trends across groups. Also in this case, planned pairwise comparisons revealed that CP infants (p = 0.009) and DD-high infants (p = 0.045) exhibited a significantly lower number of cumulative unique pose counts compared to infants with typical development.

**Fig. 6.**
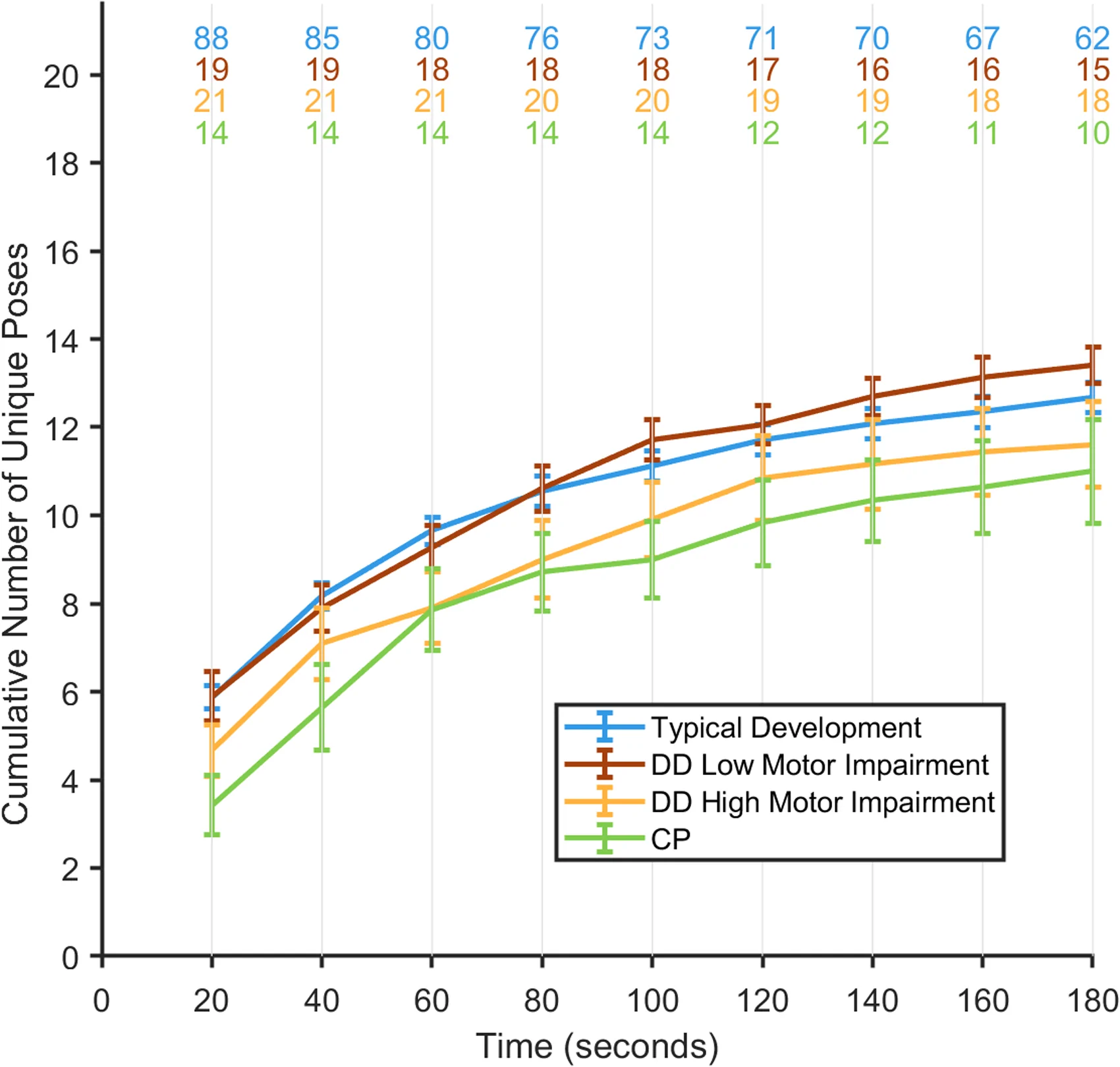
Temporal evolution of the mean cumulative unique pose count computed over increasing time windows for infants with typical development, DD with low motor impairment, DD with high motor impairment and CP. Solid lines represent group mean values, while error bars indicate the standard error of the mean. Colored numbers above each time point denote the number of infants contributing data at the corresponding time window for each group.

## IV. Discussion

The proposed approach was developed to characterize spontaneous movement organization across four levels of motor impairment in preterm infants at TEA. Significant differences emerged between infants with typical development and those with CP and DD–high motor impairment. Both groups exhibited reduced movement dynamics, reflected by lower pose transition rates and fewer unique poses. Reduced qualitative variability, measured by IQV, was primarily observed in infants with CP. These findings indicate less diverse and more constrained motor behavior compared to typically developing infants, consistent with clinical observations of reduced movement variability in infants with marked motor deficits [2], [9]. Within the DD cohort, lower gross-motor scores were associated with lower pose transition rates and movement patterns closer to those observed in CP. The graded differences across DD–low, DD–high, and CP groups suggest that the proposed pose-based representation captures variations in movement organization across increasing levels of motor impairment. The observed association between gross-motor score and both pose transition rate and IQV further supports this trend, although the magnitude of the associations was modest. Consistent patterns were also observed in the temporal evolution of the cumulative number of unique poses, which highlighted differences in repertoire expansion of the movement repertoire across severity-based subgroups. Overall, these findings indicate that the proposed pose-based representation captures clinically meaningful aspects of spontaneous movement organization.

In contrast to much of the existing literature, which primarily focuses on classification of neuromotor outcomes [18], [22], [44] this study emphasizes the extraction of interpretable features to characterize spontaneous movement across different degrees of motor impairment. By capturing changes in movement dynamics and variability, the proposed framework provides clinically meaningful insights that complement classification-based approaches.

Several limitations should be acknowledged. First, the framework relies on two-dimensional pose representations and therefore does not capture out-of-plane movements. Although three-dimensional motion analysis could provide a more complete description of infant kinematics, its application in neonatal settings remains challenging. Second, the sample size is limited, and larger multi-center studies are needed to validate the generalizability of the proposed parameters. In addition, all recordings were acquired in a controlled clinical setting preventing evaluation under more heterogeneous recording conditions. Moreover, the analysis was conducted at a single time point at TEA; longitudinal recordings could provide further insight into the developmental trajectories of spontaneous movements. Finally, the use of K-means clustering assumes a fixed number of clusters and relies on Euclidean distance to group poses, which may not fully capture subtle or complex relationships between postural configurations. However, sensitivity analyses across K = 10–30 showed that the main group-level trends were generally preserved, although dictionary granularity affected the magnitude of group differences and the sensitivity of the extracted metrics (Figs. S1 and S2, Tables S.I, S.II and S.III). Future studies could investigate alternative clustering strategies to assess whether finer postural differences can be captured.

## V. Conclusion

This study evaluated an automated framework for the objective analysis of spontaneous movements in preterm infants at term-equivalent age. By representing video recordings as temporal sequences of key poses derived from a data-driven dictionary, we extracted quantitative parameters characterizing early motor behavior. The framework revealed reduced movement activity and variability in infants with higher levels of motor impairment compared to typically developing infants. These findings support the potential of video-based representations as a scalable and non-invasive approach for characterizing early neuromotor impairment. Future work will extend this framework by incorporating additional motion descriptors, analyzing longitudinal recordings, and evaluating its robustness in less controlled environments such as home-based settings.

## Supplementary Materials

The Supplementary Materials include sensitivity analyses evaluating the robustness of the pose-based metrics across different numbers of clusters (K = 10-30).

Supplementary Materials
