# Supplementary material for: An Automated Framework for Pose-Sequence Analysis of Spontaneous Movements in Preterm Infants
Source: IEEE Open J Eng Med Biol. 2026 Sep 17;7:355–61. doi: 10.1109/OJEMB.2026.3735159 (PMC13667383; doi:10.1109/OJEMB.2026.3735159)
Supplement: Supplementary file 1 [file supp1-3735159.pdf]

Supplementary Materials

An Automated Framework for Pose-Sequence Analysis of Spontaneous Movements in Preterm Infants

Sofia Sigismondi*, Sara Uccella, Chiara Tacchino, Luca Ramenghi, Lino Nobili, Francesca Odone, Maura Casadio and Matteo Moro

To evaluate the robustness of the proposed pose-based framework to the selected number of clusters, we repeated the full pipeline for K values ranging from 10 to 30. Pose Transition Rate and IQV were recalculated and summarized across clinical groups. The main group-level trends were generally preserved, although the magnitude of group differences varied across K values (Figs. S1 and S2).

Linear regression analyses were repeated for each K value within the DD cohort. For Pose Transition Rate, β ranged from 0.005 to 0.008 and R^2 from 0.080 to 0.128 (Table S.I). For IQV, β ranged from 0.007 to 0.010 and R^2 from 0.167 to 0.234, with statistically significant associations across all K values (Table S.II).

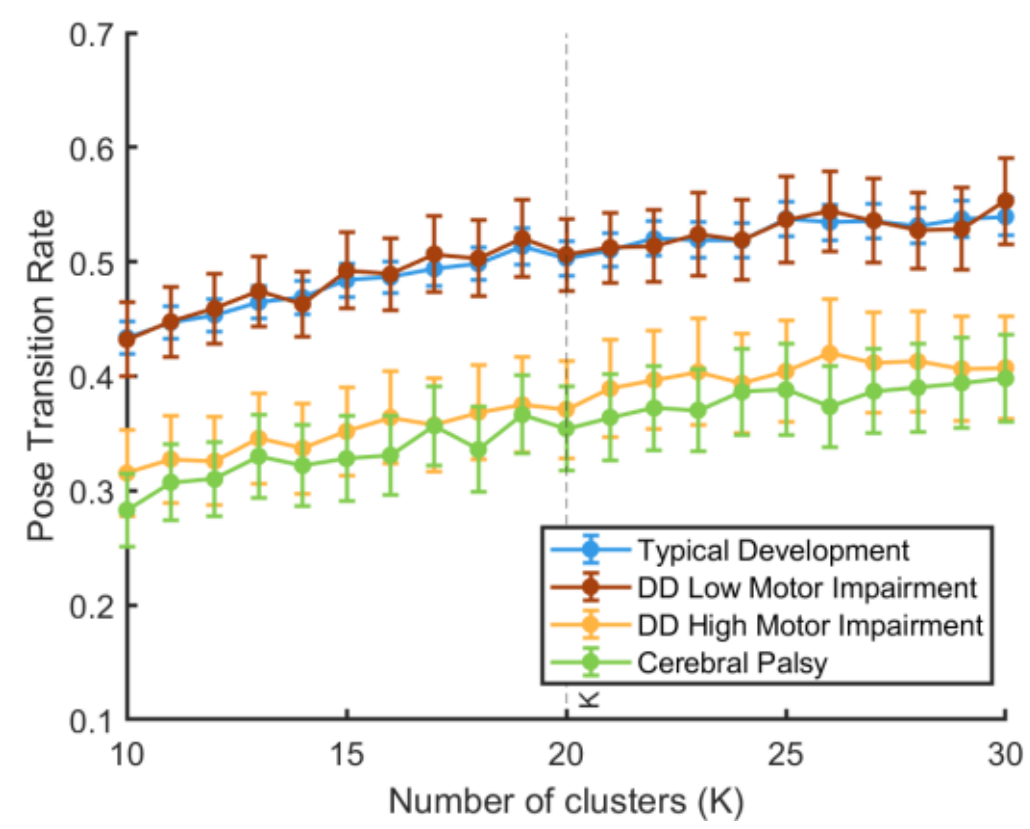

Fig. S1. The plot shows the pose transition rate across different numbers of clusters (K) for infants with typical development, DD with low motor impairment, DD with high motor impairment and CP. Points indicate the group mean and error bars indicate the standard error of the mean. The dashed vertical line indicates the value used in the main analysis (K = 20).

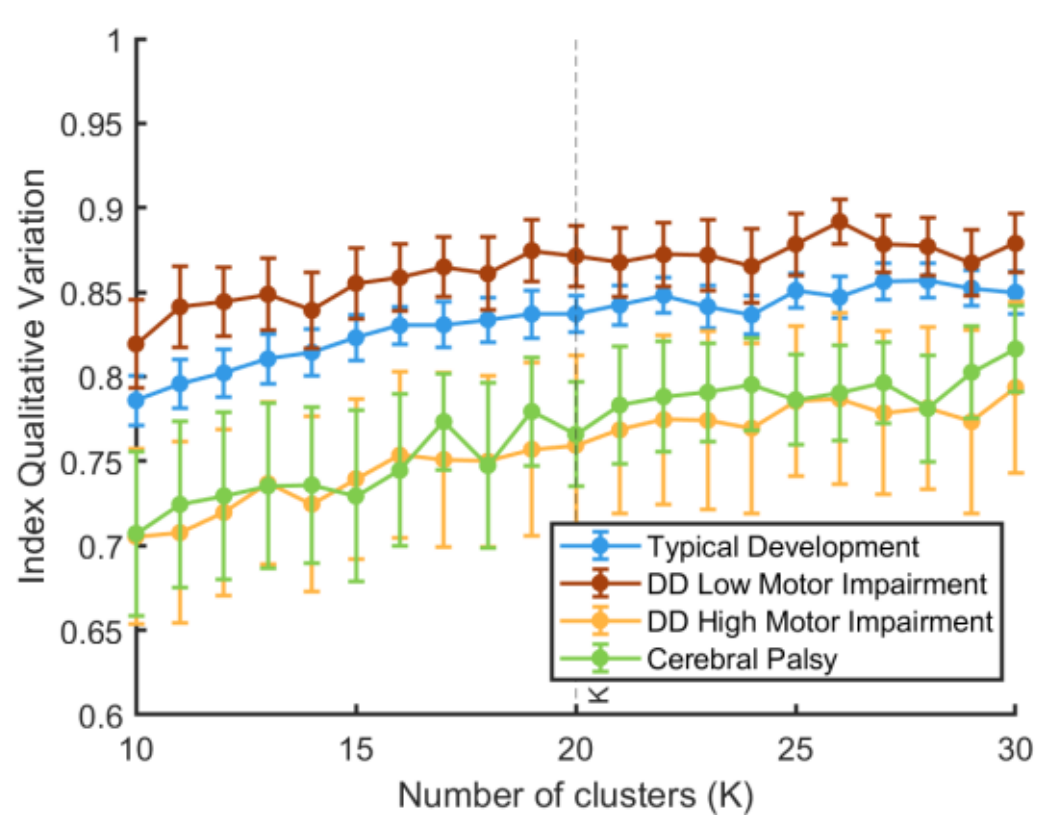

Fig. S2. The plot shows the IQV across different numbers of clusters (K) for infants with typical development, DD with low motor impairment, DD with high motor impairment and CP. Values were then summarized within each clinical group. Points indicate the group mean and error bars indicate the standard error of the mean. The dashed vertical line indicates the value used in the main analysis (K = 20).

TABLE S.I

SENSITIVITY ANALYSIS OF THE LINEAR REGRESSION BETWEEN THE GRIFFITHS GROSS-MOTOR SCORE AND POSE TRANSITION RATE ACROSS K VALUES

K	R^2	β	p
10	0.090	0.005	0.060
11	0.093	0.005	0.055
12	0.127	0.007	0.024
13	0.111	0.006	0.036
14	0.121	0.006	0.028
15	0.127	0.007	0.024
16	0.104	0.006	0.043
17	0.127	0.007	0.024
18	0.112	0.007	0.035
19	0.118	0.007	0.030
20	0.120	0.007	0.028
21	0.106	0.006	0.040
22	0.093	0.006	0.056
23	0.080	0.006	0.076
24	0.095	0.006	0.053
25	0.088	0.006	0.063
26	0.086	0.006	0.067
27	0.101	0.007	0.046
28	0.085	0.006	0.068
29	0.095	0.006	0.053
30	0.128	0.008	0.023

Linear regression analyses between the Griffiths gross motor score and pose transition rate repeated across different numbers of clusters (K), ranging from 10 to 30.

The sensitivity analysis was also extended to the Cumulative Unique Pose Count using Poisson generalized linear mixed models. Across K values, the main effects of time and group remained significant, whereas the group-by-time interaction remained non-significant. Planned comparisons showed a

generally consistent pattern, with lower cumulative unique pose counts in CP and DD–high than in typically developing infants and no consistent difference for DD–low (Table S.III).
Overall, these analyses indicate that the main findings were not driven exclusively by the selected value of K = 20. At the same time, they confirm that the number of clusters can influence the magnitude of group differences and the sensitivity of the extracted metrics, supporting the need for further optimization of dictionary granularity in future studies.

TABLE S.II
SENSITIVITY ANALYSIS OF THE LINEAR REGRESSION BETWEEN THE GRIFFITHS GROSS-MOTOR SCORE AND INDEX OF QUALITATIVE VARIATIONS ACROSS K VALUES

K	R^2	β	p
10	0.167	0.009	0.009
11	0.171	0.009	0.008
12	0.218	0.009	0.002
13	0.199	0.009	0.004
14	0.207	0.009	0.003
15	0.204	0.009	0.003
16	0.202	0.009	0.004
17	0.218	0.009	0.002
18	0.206	0.009	0.003
19	0.234	0.010	0.002
20	0.221	0.010	0.002
21	0.206	0.009	0.003
22	0.207	0.009	0.003
23	0.196	0.009	0.004
24	0.194	0.009	0.004
25	0.184	0.007	0.006
26	0.221	0.009	0.002
27	0.208	0.008	0.003
28	0.196	0.008	0.004
29	0.167	0.008	0.009
30	0.189	0.008	0.005

Linear regression analyses between the Griffiths gross motor score and Index of Qualitative Variation repeated across different numbers of clusters (K), ranging from 10 to 30.

TABLE S.III
SENSITIVITY ANALYSIS OF THE POISSON GENERALIZED LINEAR MIXED MODEL FOR CUMULATIVE UNIQUE POSES ACROSS DIFFERENT VALUES OF K

K	Poisson Generalized Mixed Model											
	Time		Group		Group-by-time		TD/DD-Low		TD/DD-High		TD/CP	
	Χ² (8)	*p*	*χ² (3)*	*p*	*χ²(24)*	*p*	*Ratio*	*p_bonf*	*Ratio*	*p_bonf*	*Ratio*	*p_bonf*
**10**	153.02	< 0.001	12.07	0.007	10.70	0.991	0.99	1.00	1.15	0.051	1.21	0.018
**11**	175.77	< 0.001	10.49	0.015	8.61	0.998	0.99	1.00	1.15	0.081	1.21	0.033
**12**	191.48	< 0.001	14.82	0.002	9.61	0.996	1.01	1.00	1.19	0.015	1.24	0.012
**13**	226.02	<0.001	14.29	0.003	9.66	0.996	0.99	1.00	1.16	0.048	1.27	0.006
**14**	248.70	< 0.001	15.33	0.002	8.87	0.998	0.98	1.00	1.22	0.009	1.24	0.021
**15**	253.14	< 0.001	11.72	0.008	10.15	0.994	0.97	1.00	1.16	0.21	1.23	0.024
**16**	296.88	< 0.001	14.04	0.003	11.64	0.984	0.98	1.00	1.20	0.021	1.25	0.018
**17**	297.08	<0.001	14.29	0.003	9.63	0.996	0.97	1.00	1.18	0.033	1.25	0.012
**18**	335.58	< 0.001	13.12	0.004	10.11	0.994	0.95	1.00	1.21	0.024	1.23	0.048
**19**	374.13	<0.001	10.84	0.013	12.16	0.978	0.96	1.00	1.17	0.087	1.23	0.042
**20**	358.9	<0.001	14.40	0.002	12.20	0.977	0.96	1.00	1.19	0.045	1.28	0.009
**21**	409.01	<0.001	9.24	0.026	12.09	0.979	0.98	1.00	1.16	0.132	1.23	0.057
**22**	420.42	< 0.001	11.84	0.008	12.30	0.976	0.99	1.00	1.21	0.018	1.24	0.042
**23**	452.13	<0.001	12.10	0.007	15.62	0.901	1.00	1.00	1.22	0.018	1.26	0.033
**24**	472.09	< 0.001	13.03	0.005	11.75	0.983	0.97	1.00	1.22	0.021	1.24	0.042
**25**	502.86	< 0.001	11.20	0.011	16.78	0.858	0.95	1.00	1.19	0.072	1.25	0.042
**26**	508.23	<0.001	14.15	0.003	14.98	0.921	0.93	1.00	1.18	0.093	1.31	0.009
**27**	546.45	<0.001	15.06	0.002	19.81	0.708	1.01	1.00	1.22	0.011	1.34	0.003
**28**	546.44	<0.001	15.07	0.002	19.80	0.708	1.01	1.00	1.22	0.033	1.34	0.003
**29**	562.56	<0.001	10.72	0.013	10.25	0.993	1.01	1.00	1.20	0.075	1.29	0.024
**30**	582.81	<0.001	11.69	0.009	10.37	0.993	0.98	1.00	1.22	0.048	1.29	0.030

Poisson generalized linear mixed models repeated across K values from 10 to 30 on the cumulative number of unique poses across the four groups: TD, typically developing; DD-Low, developmental delay with lower motor impairment; DD-High, developmental delay with higher motor impairment; CP, cerebral palsy. χ^2(df) and p refer to Wald omnibus tests of fixed effects. Ratios indicate pairwise ratios of estimated marginal means, calculated as TD divided by the corresponding impaired group. *p_bonf* denotes Bonferroni-adjusted p-values for the comparisons between TD and each impaired group.
